# Analyzing the associations between tertiary lymphoid structures and postoperative prognosis, along with immunotherapy response in gastric cancer: findings from pooled cohort studies

**DOI:** 10.1007/s00432-024-05672-y

**Published:** 2024-03-22

**Authors:** Haoxin Peng, Xiangrong Wu, Cheng Zhang, Yueting Liang, Siyuan Cheng, Honglang Zhang, Lin Shen, Yang Chen

**Affiliations:** 1 Department of Gastrointestinal Oncology, Key Laboratory of Carcinogenesis and Translational Research (Ministry of Education), Peking University Cancer Hospital & Institute, Beijing, China; 2 Department of Oncology, Shanghai Medical College, Fudan University, Shanghai, China; 3 Department of Radiation Oncology, Peking University Cancer Hospital & Institute, Beijing, 100142 China; 4 Department of Tumor Chemotherapy and Radiation Sickness, Peking University Third Hospital, Beijing, China; 5 Department of Clinical Medicine, Nanshan School, Guangzhou Medical University, Jingxiu Road, Panyu District, Guangzhou, China; 6 Department of Gastrointestinal Oncology, State Key Laboratory of Holistic Integrative Management of Gastrointestinal Cancers, Beijing Key Laboratory of Carcinogenesis and Translational Research, Peking University Cancer Hospital & Institute, Beijing, China

**Keywords:** Tertiary lymphoid structure, Gastric cancer, Overall survival, Immunotherapy

## Abstract

**Background:**

The clinical significance of tertiary lymphoid structure (TLS) in gastric cancer (GC) was uncertain.

**Methods:**

A systematic search was performed in public databases for eligible studies as of April 2, 2023. Meta-analyses were performed to interrogate the associations between TLS levels and prognosis and immunotherapy response of GC. Bioinformatic analyses based on the nine-gene signature of TLS were further conducted to capture the biological underpinnings.

**Results:**

Eleven studies containing 4224 GC cases were enrolled in the meta-analysis. TLS levels positively correlated with smaller tumor size, earlier T stage and N stage. Moreover, higher TLS levels were detected in diffuse and mix subtypes of GC (*P* < 0.001). Higher TLS levels strongly predicted favorable postoperative overall survival of GC, with HR of 0.36 (95%CI 0.26–0.50, *P* < 0.001) and 0.55 (95%CI 0.45–0.68, *P* < 0.001) of univariate and multivariate Cox analysis, respectively. Higher TLS levels were also in favor of the treatment response of anti-PD-1 inhibitors as later-line therapy of GC. TLS levels positively correlated with immune effector cells infiltration, diversity and richness of T cell receptor and B cell receptor repertoire, immune checkpoint genes expression, and immune-related genes mutation of GC in the TCGA-STAD cohort, representing higher immunogenicity and immunoactivity. Moreover, moderate accuracy of TLS levels in predicting benefit from anti-PD-1 inhibitors in the PRJEB25780 cohort was also validated (AUC 0.758, 95%CI 0.583–0.933), higher than the microsatellite instability-score and Epstein-Barr virus status.

**Conclusions:**

TLS levels demonstrated potential in predicting the postoperative prognosis and immunotherapy response of GC.

**Supplementary Information:**

The online version contains supplementary material available at 10.1007/s00432-024-05672-y.

## Introduction

Gastric cancer (GC) is a major cause of cancer-related mortality globally (Sung et al. 2021), with exceptionally high disease burden in East Asia (Etemadi 2017). GC is featured by high heterogeneity at histological, cellular, (epi)genomic, and proteomic levels, accompanied by distinct clinical outcomes (Wadhwa et al. 2013; Ge et al. 2018). Despite good response to treatment in early-stage GC, advanced GC is highly aggressive, with a median overall survival (OS) time of around 10 months (Smyth et al. 2020). In recent years, immunotherapy has revolutionized the treatment landscape of advanced GC and significantly extended patients’ survival (Li et al. 2021a). However, only a limited population benefited from immunotherapy, and a wide variation of response rates was reported, calling for better biomarkers for stratified treatment (Janjigian et al. 2021; Shitara et al. 2020).

Proteomic markers such as PD-L1 expression (Kim et al. 2018) and molecular markers such as microsatellite instability (MSI) (Kwon et al. 2021) have been established as potential biomarkers of prognosis and responses to immune checkpoint inhibitors (ICIs), whereas findings between different clinical trials were inconsistent. Recently, components in the tumor microenvironment (TME) of GC, such as the contents (Ren et al. 2021) and spatial location (Chen et al. 2022) of different cell types, and specialized structures like tertiary lymphoid structure (TLS) (Yu et al. 2022), have received increasing attention for they could affect prognosis and immunotherapy efficiency.

Composed of cellular aggregates in non-lymphoid organs under inflammatory conditions like infection and tumor, TLSs show analogical functional and structural features with lymph nodes (Schumacher and Thommen 2022). Mature TLS is characterized by the B-cell zone that involves the germinal center and is surrounded by the follicular helper T cells. The T-cell zone containing dendritic cells and high endothelial venules is also crucial to TLS (Sautès-Fridman et al. 2019). TLSs signify privileged regions for immune cell maturation and antigen presentation, serving as the crucial milieu for anti-tumor immunity. Emerging evidence indicated that TLS presence strongly correlated with higher immunoreactivity and better clinical outcomes of GC. For instance, Li and colleagues reported that TLS presence was indicative of favorable OS based on a cohort containing 63 GC cases (Li et al. 2020), and Yin et al. further showed that TLS was a promising predictor for longer survival of Epstein-Barr Virus (EBV)-associated GC (Yin et al. 2022). Moreover, Jiang et al. proposed that TLS positively correlated with superior response of ICIs based on a cohort containing 13 GC samples (Jiang et al. 2022). However, some studies were limited by small sample sizes or specific GC subtypes, thus may lack generalizability to some extent. Moreover, associations between TLS and prognosis and therapeutic sensitivity of GC are controversial, probably because samples from different ethnicities and different detecting approaches for TLS were applied.

Consequently, we conducted a meta-analysis to clarify the prognostic and predictive values of TLSs in GC. Simultaneously, we performed bioinformatic validation to capture the biological underpinnings by TLS-related gene signature in external cohorts. Our comprehensive analyses provided the latest evidence for the relationships between TLS and GC, probably conveying a powerful biomarker for clinical practice.

## Methods

### Guidance and protocol

The present study was conducted based on Preferred Reporting Items for Systematic Reviews and Meta-Analysis (PRISMA) (Liberati et al. 2009), and the protocol was registered at the Prospective Register of Systematic Reviews (PROSPERO ID CRD42023413227).

### Search strategy

Two authors (H.X.P. and X.R.W.) independently searched publicly available databases, including the Cochrane Library, Embase, PubMed, and Web of Science, to retrieve suitable studies before April 2, 2023. The references of identified articles were also reviewed to seek potential research. The search strings are presented in Supplementary Table 1.

### Included and excluded criteria

Studies were regarded eligible if: (1) focusing on GC populations; (2) examining TLSs in situ of tumor samples by immunohistochemistry (IHC) or hematoxylin–eosin (H&E) staining; (3) evaluating the associations between TLSs and survival or therapeutic response of GC; (4) publishing in English with available full-text. Studies were excluded if: (1) sample size < 10; (2) comments, conference abstracts, or letters to the editor; (3) outcome data could not be obtained or estimated.

### Study selection

Independent authors H.X.P. and X.R.W. screened titles and abstracts to obtain eligible studies, the full texts of which were further reviewed. Disagreements were addressed via discussion with senior investigators (Y.C.).

### Data extraction

Researchers (H.X.P. and X.R.W.) independently utilized standardized forms to collect data, such as sample size, clinicopathologic characteristics, and TLS location and detecting approaches. Outcome measures were also extracted, including hazard ratio (HR) with corresponding 95% confidence interval (CI) and number of responders/non-responders to ICIs.

### Quality assessment

The Newcastle–Ottawa Scale (NOS), with scores ranging from 0 to 9, was exploited to estimate the quality of the included study. Two independent authors (H.X.P. and X.R.W.) performed the workflow, and differing opinions were resolved by consensus. A study with a NOS score greater than 6 was determined high-quality.

### Evaluating correlations between TLSs and clinicopathologic parameters of GC

Clinicopathologic data of GC samples, including age, tumor size, T stage, N stage, pTNM stage, and differentiation status, were extracted and re-classified into a high and low group of each parameter. Specifically, age of 50, tumor size of 5 cm, T1 + T2/T3 + T4 stage, N0/N1 stage, I + II/III + IV pTNM stage**,** and poor/moderate or well differentiation were used as cut-off values. Then, chi-square and Fisher’s exact tests were exploited to compare differences between groups with or without TLSs.

### Data synthesis

Statistical analyses were performed on Stata (version 15) and R (version 4.3.1) software. Considering the between-study differences in detecting and quantifying TLS, TLS levels were utilized uniformly to report the findings. For outcome measures of prognosis, HRs and corresponding CIs were pooled. For ICIs response, outcome measures were expressed and pooled as odds ratio (ORs) and 95%CIs. Inter-study heterogeneity was estimated by *I*^2^ statistic, and it was considered notable if *I*^2^ ≥ 50% (Higgins and Thompson 2002). The random-effects model was adopted if substantial heterogeneity was observed. Otherwise, the fixed-effects model was utilized. Statistical significance was defined at *P* < 0.05.

### Publication bias and sensitivity analyses

The funnel plot test, Begg’s test, and Egger’s test were employed to assess publication bias, which was further tested and adjusted by the trim-and-fill method (Duval and Tweedie 2000). Sensitivity analysis was performed by removing each study one by one.

### Biological validation of TLS signature

The genomic profiles, mRNA expression, T-cell receptor (TCR) and B-cell receptor (BCR) repertoire, and clinical characteristics data of GC samples (*n* = 443) from the TCGA-STAD cohort were downloaded from the UCSC Xena (https://xena.ucsc.edu/) database. The single-sample Gene Set Enrichment Analysis (ssGSEA) approach was adopted to calculate the enrichment scores of TLS by the nine-gene signature (CCL19, CCR7, CETP, CXCL10, CXCL11, CXCL13, CXCL9, LAMP3, SELL) as previously reported (Cabrita et al. 2020; Hou et al. 2022).

As for immune infiltration estimation, enrichment scores of 29 immune signatures were computed via the ssGSEA method (He et al. 2018), and the abundance of 22 immune cell lineages was quantified through the CIBERSORT algorithm (Newman et al. 2015) based on bulk RNA-seq data.

### Predicting ICIs efficacy of the TLS signature

The clinical and transcriptomic data were collected from the PRJEB25780 cohort, in which metastatic GC patients (*n* = 61) were treated with pembrolizumab monotherapy as later-line therapy (Kim et al. 2018). Forty cases (65.6%) had more than two sites of metastasis and nearly half of them had previously undergone second-line therapy. Twenty-eight (45.9%) and twelve (19.7%) patients had PD-L1 combined positive score of more than 1 and 5, respectively. Moreover, 6 patients were tested to be EBV positivity, and 7 patients held MSI-H status. The TLS-score represented by the nine-gene signature was calculated for each sample in the same manner to discover the predictive effects of TLS-score on ICIs efficacy.

### Statistical analysis

Mann–Whitney *U* test and chi-square test were applied to compare categorical and continuous data, respectively. Correlation analysis was conducted via Spearman’s test. Visualization of survival differences was generated by Kaplan–Meier curves and tested through the log-rank test. The threshold of statistical significance was set as *P* < 0.05.

## Results

### Study collection and characteristics

**Fig. 1:**
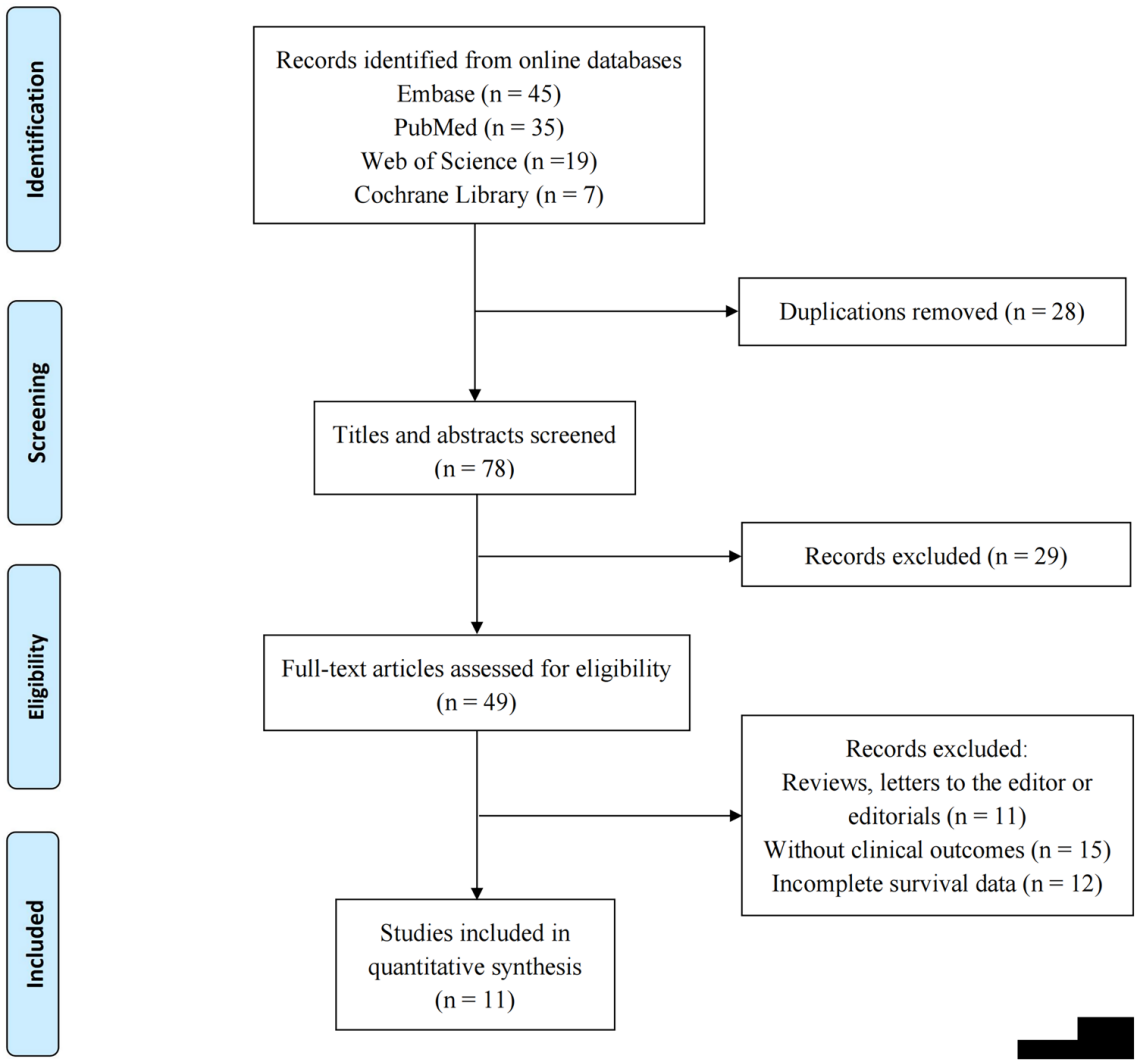
The flow diagram of searching and selecting study process

As presented in the PRISMA workflow (Fig. 1), 106 records were identified from public databases in the initial literature search. After carefully screening titles and abstracts, and subsequent full-text review, 11 studies incorporating 4224 GC samples were ultimately enrolled (Yu et al. 2022; Li et al. 2020; Yin et al. 2022; Jiang et al. 2022; Li et al. 2023; Kemi et al. 2023; Mori et al. 2022; Cheng et al. 2021; Mori et al. 2021; Yamakoshi et al. 2021; He et al. 2020). Baseline information and major characteristics are shown in Table 1.

**Table 1:** Major characteristics of the included studies

References	Publication year	Country	Sample size	pStage	TLSs detection approaches	TLSs location	Cut-off criteria	Follow-up time (months) median (range)	Endpoint
Zhe Li (2023)	2023	America	685	I ~ IV	H&E	Global	TLS score: 1 ~ 3	NA	OS
YiXin Yin (2022)	2022	China	148	I ~ IV	H&E	Intratumoral/peritumoral	Positive/negative	NA	OS; response to ICI
Niko Kemi (2023)	2023	Finland	721	NA	H&E	Global	Diameter; density	28 (0 ~ 432)	OS
Quan Jiang (2022)	2022	China	292	I ~ IV	IHC	Global	Immature/mature/integral	NA	OS
Jishang Yu (2022)	2022	China	118	I ~ III	IHC	Global	Density: high/low	NA	OS, DFS
Takuya Mori (2022)	2022	Japan	19	I ~ IV	IHC	Global	Density: high/low	NA	Response to ICI
Na Cheng (2021)	2021	China	804	I ~ IV	IHC	Global	Positive/negative	22.1 (1 ~ 99)	OS
Takuya Mori (2021)	2021	Japan	261	I ~ IV	IHC	Global	Density: rich/poor	NA	OS; response to ICI
Yoshihito Yamakoshi (2021)	2021	Japan	199	I ~ IV	IHC	Global	Density: high/low	49 (1–92)	OS
Qing Li (2020)	2020	China	63	I ~ III	IHC	Global	Density: high/low	NA	OS
Wenting He (2020)	2020	China	914	I ~ III	IHC	Tumor center/invasive margin	Number; density	NA	OS

*H&E* hematoxylin–eosin, *IHC* immunohistochemistry, *ICI* immune-checkpoint inhibitor, *NA* not available, *OS* overall survival, *TLS* tertiary lymphoid structure

The majority of studies were from East Asian countries (*n* = 9, 82%), reflecting the relatively high disease burden of GC. Among the 11 included studies, 8 studies reported the associations between TLS levels and postoperative prognosis of GC, 2 studies additionally reported the predictive values of TLS on immunotherapy response, and the rest one only reported the predictive significance of TLS on immunotherapy (Table 1). Two studies assessed the TLS location within tumor tissue, i.e., intra-/peri-tumor, while the remaining applied global estimation. Seven out of eleven studies enrolled stage IV GC patients, and the total number of stage IV GC cases included in the meta-analysis was 151, accounting for 3.6% of the overall population. Eight studies used IHC to detect TLS, while the residuals applied H&E staining. The NOS scores of the enrolled studies ranged from 5 to 8, with eight studies achieving high-quality (73%) (*Supplementary Table 2*).

### Associations between TLS levels and clinicopathologic features

Higher TLS levels positively correlated with smaller tumor size (OR 0.674, 95%CI 0.505–0.901, *P* = 0.008), earlier T stage (OR 0.187, 95%CI 0.096–0.366, *P* < 0.001) and N stage (OR 0.758, 95%CI 0.608–0.944, *P* = 0.013) (Table 2). Higher TLS levels also correlated with a trend of earlier pTNM stage whereas without statistical significance. Interestingly, diffuse (*P* < 0.001) and mix (*P* < 0.001) subtypes of GC were positively associated with higher TLS levels. No significant associations between age, gender or differentiation status and TLS levels were observed.

**Table 2:** Associations between tertiary lymphoid structure and clinicopathological characteristics

Parameters	Number of studies	Number of cases	OR (95%CI)	χ2 value	*P* value	Relationship with TLS
Age	4	1899	1.024 (0.722–1.453)	0.018	0.895	Non-significance
Gender	5	667	1.098 (0.936–1.287)	1.319	0.251	Non-significance
Tumor size	2	824	0.674 (0.505–0.901)	7.134	0.008	Negative
T stage	3	388	0.187 (0.096–0.366)	27.213	< 0.001	Negative
N stage	5	1219	0.758 (0.608–0.944)	6.127	0.013	Negative
pTNM stage	7	2744	0.921 (0.808–1.050)	1.511	0.219	Non-significance
Differentiation status	5	1538	1.038 (0.868–1.240)	0.164	0.685	Non-significance
Lauren class (Intestinal vs. Diffuse)	2	2138	1.647 (1.385–1.959)	31.977	< 0.001	Positive
Lauren class (Intestinal vs. Mix)	2	1021	2.675 (1.722–4.156)	20.343	< 0.001	Positive
Lauren class (Diffuse vs. Mix)	2	1333	1.624 (1.050–2.513)	4.818	0.028	Positive

*CI* confidence interval, *OR* odds ratio, *pTNM* pathological TNM, *TLS* tertiary lymphoid structure

### Prognostic effects of TLS levels on GC survival

**Fig. 2:**
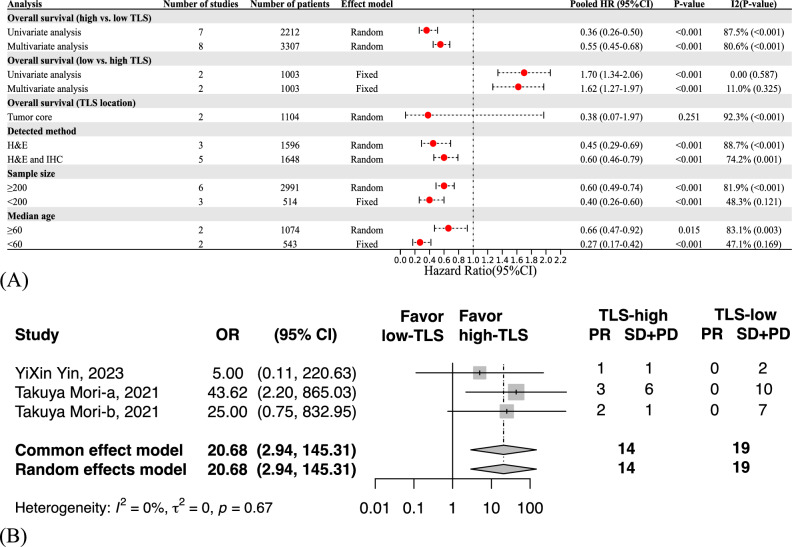
Clinical significance of tertiary lymphoid structures (TLSs) in gastric cancer (GC). The postoperative prognostic effects of TLSs on overall survival of GC, stratified by analytical methods, TLSs location, detected methods, sample size, and median age of the included cases (**A**). The predictive effects of TLSs on immunotherapy response of GC (**B**). *OR* odds ratio; *PD* progressive disease; *PR* partial response; *SD*, stable disease

Meta-analysis was conducted to pool HRs and corresponding CIs for OS *(*Fig. 2A*, *Supplementary Fig. 1). In the univariate analyses, TLS levels correlated with significantly better OS of GC (high vs. low: HR 0.36, 95%CI 0.26–0.50, *P* < 0.001; low vs. high: HR 1.70, 95%CI 1.34–2.06, *P* < 0.001). However, high heterogeneity was observed, likely caused by heterogeneous GC subtypes and different TLS detecting methods. The favorable prognostic effects of TLS remained significant in the multivariate analyses (high vs. low: HR 0.55, 95%CI 0.45–0.68, *P* < 0.001; low vs. high: HR 1.62, 95%CI 1.27–1.97, *P* < 0.001). Details of the covariates used for multivariate analysis of each study are summarized in Supplementary Table 3.

Subgroup analyses were also performed according to detection methods, sample size, and median age of included patients. The prognostic effects of TLS levels on GC survival stayed salient across different subgroups while slightly attenuated in combined H&E staining and IHC detection subgroup and sample size greater than 200 subgroup. Interestingly, TLS presence was a strong and favorable prognosticator of younger (HR 0.27, 95%CI 0.17–0.42, *P* < 0.001) than elder (HR 0.66, 95%CI 0.47–0.92, *P* = 0.015) GC patients. Only two studies assessed the prognostic effects of intratumoral TLS, while insignificant association (HR 0.38, 95%CI 0.07–1.97, *P* = 0.251) and high heterogeneity (*I*^2^ = 92.3, *P* < 0.001) were found, possibly attributed to limited cases. Collectively, TLS was a strong and favorable postoperative prognosticator of GC.

### Publication bias and sensitivity analyses

Despite Begg’s test result being insignificant (*P* = 0.273), the Funnel plot was not symmetrical, and Egger’s test indicated the existence of publication bias (*P* = 0.005). The trim-and-fill approach was further employed to test and adjust the publication bias. Results demonstrated the possibility of publication bias (HR 0.72, 95%CI 0.51–1.01), potentially attributed to the high heterogeneity of the included studies (Supplementary Fig. 2). The leave-one-out analysis demonstrated an insignificant impact on the merged effects after excluding any of the enrolled research, implying the stability of the finding.

### Predictive effects of TLS levels on ICIs response

Three studies with 33 GC patients receiving anti-PD-1 therapy were enrolled. Two cohorts enrolled GC patients with nivolumab as a third-line or later treatment (Mori et al. 2022; Mori et al. 2021), and the rest did not provide more details (Yin et al. 2022). A total of 6 patients reached partial response (PR), while the residuals were defined as stable disease (SD) and progressive disease (PD) (Schwartz et al. 2016). Meta-analysis showed that higher TLS levels favored ICIs response (OR 20.68, 95%CI 2.94–145.31, *P* = 0.002), and broad CI may be attributed to the limited sample size (Fig. 2B).

### TLS signature in the TCGA-STAD database

The main dilemma for studying TLS lies in the absence of a criterion for detecting and quantifying. Traditional approaches such as IHC and H&E staining were time-consuming and prone to subjective bias. The gene signature of TLS derived from transcriptomic data, primarily representing T cells and B cells in TLS, has been recently proposed and shown to be effective in quantifying TLSs (Sautès-Fridman et al. 2019). The 9-gene TLS signature has been adopted in melanoma (Cabrita et al. 2020), ovarian (Hou et al. 2022), and lung cancer (Feng et al. 2021), conveying notable predictive and prognostic implications.

**Fig. 3:**
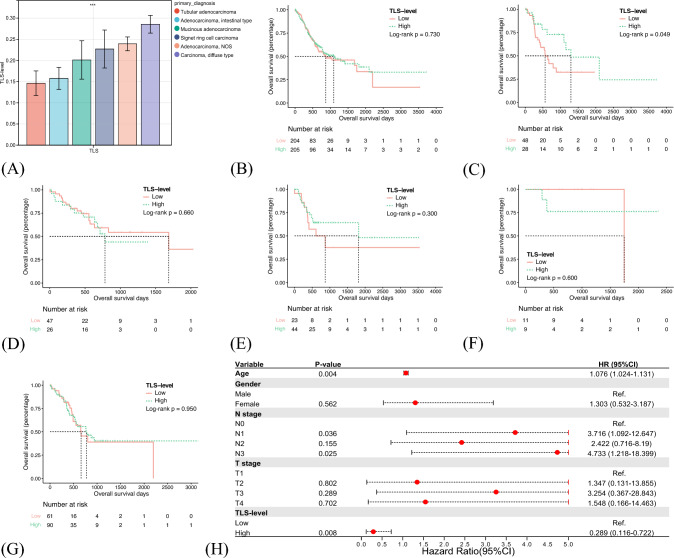
The tertiary lymphoid structures (TLS)-level spectrums of different gastric cancer (GC) subtypes and their prognostic effects. TLS levels varied significantly among different pathological subtypes of GC (**A**). Overall survival differences between high/low TLS-level groups of overall GC (**B**), intestinal GC (**C**), tubular GC (**D**), diffuse GC (**E**), mucinous GC (**F**), and not otherwise specified adenocarcinoma (**G**). The prognostic value of TLS levels on intestinal GC, as evaluated by multivariate Cox regression analysis (**H**). *NOS* not otherwise specified

Firstly, considering the heterogeneity of GC, we compared the TLS levels between different GC subtypes (Fig. 3A). Diffuse GC (DGC) conveyed the highest TLS levels, while tubular not otherwise specified. We additionally compared the TLS levels among different molecular subtypes of GC, including EBV-infected, MSI, genomically stable, and chromosomally unstable tumors. Results showed that the EBV-infected subtype held the highest TLS levels, while the chromosomally unstable subtype demonstrated the lowest, conforming to the pathological mechanism associated with TLS formation (Supplementary Fig. 3A). GC samples were then divided into TLS-high/low groups upon the median value of TLS levels as a cutoff point. Insignificant OS difference was observed between TLS-high and TLS-low groups of overall GC (Log-rank *P* = 0.73) (Fig. 3B). Subgroup analysis showed that a high TLS level indicated longer OS of intestinal GC (Log-rank *P* = 0.049) (Fig. 3C–G). Multivariate Cox regression analysis adjusting for age, gender, T stage, and N stage further conveyed that TLS level was an independent prognostic factor of intestinal GC (HR 0.289, 95%CI 0.116–0.722, *P* = 0.008) (Fig. 3H). A trend of favorable prognosis in the TLS-high group of DGC was also shown, despite without statistical significance. Moreover, insignificant associations between TLS levels and prognoses of different molecular GC subtypes were observed (Supplementary Fig. 3B–E).

**Fig. 4:**
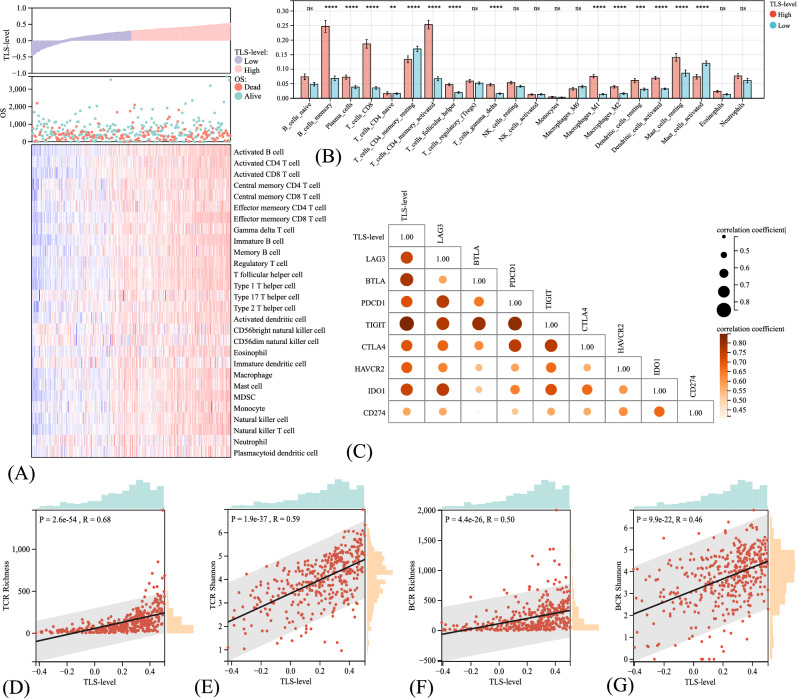
Immune landscape differences between high and low tertiary lymphoid structure (TLS)-level groups in the TCGA-STAD dataset. The distribution of TLS-level, overall survival status, and corresponding immune profiles, as evaluated by the ssGSEA method (**A**). The immune infiltration differences between high and low TLS-level groups, as evaluated by the CIBERSORT approach (**B**). Correlation heatmap demonstrating the associations between TLS levels and expression of major immune-checkpoint genes (**C**). Scatter plots indicating the associations between TLS levels and richness and diversity of T-cell receptor (**D**, **E**) and B-cell receptor (**F**, **G**) repertoire. *P* values of the Wilcoxon *t* test, **P < 0.01; ***P < 0.001; ****P < 0.0001; *ns* non-significant

We subsequently deciphered the TME landscapes concerning TLS levels, aiming at seeking out biological underpinnings responsible for the prognostic values of TLS. Significantly different infiltrated features between high and low TLS-level groups were found. Infiltration of major immune effector cells, including CD8 + T cells, CD4 + T cells, memory B cells, and natural killer cells, were significantly higher in the high TLS-level group than in low ones, indicating an “immune-hot” TME (Fig. 4A, B). TLS-score also positively correlated with the richness and diversity of TCR (Fig. 4D, E) and BCR repertoire (Fig. 4F, G) (R > 0.45, *P* < 0.001), suggesting higher antigen presentation function. Moreover, the TLS levels were positively associated with the expression levels of major immune checkpoint genes, including LAG3, TIGIT, CD274, and BTLA (Fig. 4C), implying potential benefit from immunotherapy.

**Fig. 5:**
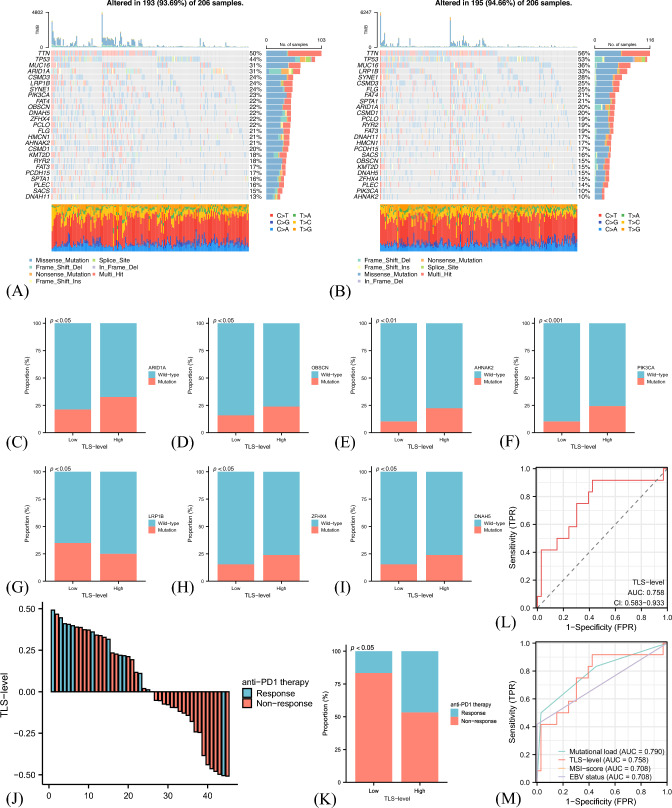
Genomic mutation landscapes concerning tertiary lymphoid structure (TLS) levels and their predictive values of immunotherapy response. Gene mutation spectrum differences between low and high TLS-level groups (**A**, **B**). Mutational frequencies of ARID1A (**C**), OBSCN (**D**), AHNAK2 (**E**), PIK3CA (**F**), LRP1B (**G**), ZFHX4 (**H**), and DNAH5 (**I**) differed significantly between high and low TLS-level groups. Response rate differences to anti-PD-1 therapy between low and high TLS-level groups in the PRJEB25780 cohort (J-K). Predictive accuracy of immunotherapy benefits of TLS-level (L) and other established biomarkers (M). *P* values of the chi-square tests between different groups

The mutation landscapes upon TLS levels were also depicted, resulting in significantly different genetic spectrums (Fig. 5A, B). Among the top 25 mutant genes, the mutated frequencies of ARID1A (*P* < 0.05) (Li et al. 2019), OBSCN (*P* < 0.05) (Liu et al. 2021), and AHNAK2 (*P* < 0.01) (Zheng et al. 2021), which were previously reported to correlate with higher immunogenicity, were significantly higher in the TLS-high group (Fig. 5C–I). Moreover, the mutant frequency of PIK3CA (*P* < 0.05) was significantly higher in the TLS-high group.

Collectively, TLS levels positively correlated with immune effector cells infiltration, TCR and BCR repertoire diversity and richness, immune checkpoint genes expression, and immune-related genes mutation of GC, signifying potential benefit from immunotherapy.

### Validation of the predictive effects of TLS in the PRJEB25780 cohort

We then verified whether TLS signature could predict ICIs benefits in an external cohort. A total of 7 (7 out of 15) and 5 (5 out of 30) responders to anti-PD-1 inhibitors in the TLS-high and TLS-low groups were found, respectively. The proportion of responders was strikingly higher in the high than low TLS-score group (*P* < 0.05) (Fig. 5J, K). TLS levels also showed moderate accuracy in predicting benefit from immunotherapy (AUC 0.758, 95%CI 0.583–0.933) (Fig. 5L), higher than the MSI-score (AUC 0.708, 95%CI 0.563–0.854) and EBV-status (AUC 0.708, 95%CI 0.563–0.854) while slightly lower than mutation load (AUC 0.790, 95%CI 0.632–0.949) (Fig. 5M). In brief, the TLS levels showed potential as a biomarker for immunotherapy response of GC.

## Discussion

Through a comprehensive meta-analysis of 11 studies containing 4,224 GC cases, we pinpointed that TLS correlated with favorable prognosis and ICIs sensitivity of GC. Biological validation in the TCGA-STAD and PRJEB25780 cohorts further corroborated that TLS presence signified higher immunoactivity in TME.

The prognosis of GC is known to be affected by tumor- and host-correlated characteristics, such as age, pTNM stage, and histologic subtypes. Consequently, we first interrogated the relationships between TLS levels and clinicopathologic features. Results showed that higher TLS levels correlated with smaller tumor size and earlier T and N stages, consistent with the findings in breast cancer (BC) (Wang et al. 2022) and lung cancer (Rakaee et al. 2021). Interestingly, higher TLS levels were discovered in diffuse and mix than intestinal subtypes of GC. Since TLS is distinguished by B-cell enriched regions, a recent single-cell atlas also documented significantly higher B-cell infiltration as a salient feature of DGC (Kumar et al. 2022). Wang et al. also reported that TLS presence predicted higher tumor-infiltrating lymphocyte (TIL) levels of BC by meta-analysis (Wang et al. 2022). Nonetheless, we could not assess whether TLS levels correlate with higher infiltration of TILs in meta-analysis due to the nature of the data.

Systematical meta-analysis indicated that higher TLS levels strongly predicted favorable OS of postoperative GC, with HR of 0.36 and 0.55 of univariate and multivariate Cox analysis, respectively. Sensitivity analyses further validated the stability and robustness of our findings. Noteworthy, high heterogeneity was found between the included studies. Thus, subgroup analyses stratified by the TLS detecting methods, sample size, and median age of the included cases were carried out to interrogate potential heterogeneity. Strikingly, the prognostic value of TLS remained significant across subgroups and was more notable in younger than elder GC cases. The heterogeneity decreased as expected, whereas it remained modest. Moreover, meta-analysis implicated that GC patients with high TLS levels significantly benefited from anti-PD-1 inhibitors as later-line therapy than those with low TLS levels. However, considering the limited included cases and retrospective design of studies, the predictive effects of TLS on ICIs sensitivity need to be interpreted cautiously.

The maturation degree of TLS is postulated to affect its clinical significance by recent work. For instance, mature TLS with GC predicted significantly longer survival than total TLSs, whereas such prognostic effect attenuated when the formation of GC was damaged (He et al. 2023; Ling et al. 2022). Meanwhile, the prognostic value of TLS varied by its location in the tumor tissue. Intratumor TLS seemingly demonstrated a stronger prognostic effect than peritumor TLS, whereas findings were inconsistent between different cancer types (Sofopoulos et al. 2019; Li et al. 2021b). There is evidence that TLS levels remarkably varied from early to advanced stage of cancer (Sautès-Fridman et al. 2019). Additionally, studies showed that TLS levels significantly attenuate in the metastatic sites compared to the primary tumors, and can even be absent (Lee et al. 2019). And patients who hold TLS both in the primary tumor and metastatic site exhibit superior prognosis (Cipponi et al. 2012). However, we could not perform comprehensive subgroup analyses on the maturation degree or spatial location of TLS due to the nature of data. Therefore, future studies characterizing TLS with compositional, spatial, and functional details are imperative and encouraged.

Considering the unique puzzles in identifying and quantifying TLSs via traditional methods like H&E staining and IHC, we further validated the findings from meta-analysis and discovered the underlying biological underpinnings via TLS-relevant gene signature, which has been verified in multiple cancer types (Cabrita et al. 2020; Hou et al. 2022; Feng et al. 2021). Strikingly, higher TLS levels were found in DGC than in other subtypes, in congruence with the meta-analysis. Despite without significance, a trend of better prognosis was also observed in DGC individuals with higher TLS levels. Moreover, TLS level was proven to be an independent prognostic factor of intestinal GC.

The TME landscapes concerning TLS levels were subsequently depicted. Higher immune infiltration of major immune effector cells, such as T and B lymphocytes and natural killer cells, were observed in the high TLS-level group than in low ones, indicative of an “immune-hot” TME. Future studies integrating TLS levels and immune infiltration features within TME may offer a more comprehensive and robust prognosticator. Interestingly, higher mast cell (MC) infiltrates were found in the TLS-low group. Evidence showed that MCs could stimulate regulatory T cells to facilitate GC progression (Lv et al. 2023). Studies also reported that inhibition of the degranulation of MC attenuated the development of GC, signifying a potential target (Gunjigake et al. 2021). We also parsed the relationships between the TLS levels and the diversity and richness of the immune repertoire, which represents the strength and breadth of immune responses and acts a paramount role in anti-tumor immunity (Jiang et al. 2019). Higher diversity and richness of TCR and BCR were discovered in the TLS-high group, representing higher antigen presentation function.

Higher TLS levels also correlated with upregulated immune checkpoint genes expression. This may also partly explain why ICIs boost strong antitumor immunity in cancers with enriched TLSs (Petitprez et al. 2020). Intriguingly, ICIs could also instigate TLS formation. For instance, Sarah et al. reported the accumulation of TLS-correlated B cells in responders after neoadjuvant ICIs of melanoma (Helmink et al. 2020). Moreover, the genetic portraits significantly differed between different TLS-level groups. Elevated mutational frequencies of several genes that correlated with immune infiltration were observed in the TLS-high group. For example, a higher mutation rate of ARID1A, a tumor suppressor gene that is relevant to the MSI feature of cancers (Mullen et al. 2021), was found in the TLS-high group. ARID1A-mutated GC held higher TMB and PDL1 levels and favored higher immune cell infiltrates (Li et al. 2019). In brief, high TLS levels represent high immunogenicity and immunoactivity, possibly driving benefits from immunotherapy.

Eventually, we interrogated whether TLS levels could predict ICIs response in the PRJEB25780 cohort, in which GC patients received later-line pembrolizumab monotherapy. Intriguingly, more responders were identified in the TLS-high group than the low ones. Moderate accuracy in predicting benefit from ICIs of TLS-level was presented (AUC > 0.75), higher than the MSI and EBV-status, which are established biomarkers indicative of immunotherapy response (Bai et al. 2022; Yu et al. 2022).

The present work firstly and comprehensively offered substantial evidence for the clinical significance of TLS in GC by meta-analysis and biological validation. Meanwhile, several limitations should be noted. First, different scoring approaches and thresholds in evaluating high/low TLS levels were utilized in different studies. However, we could not perform corresponding subgroup analyses due to unavailable data, probably leading to bias. Second, the pooled sample size for discovering and validating the predictive effect of TLS on ICIs response was limited, thus may lack robustness. Third, high heterogeneity among studies caused potential publication bias in the meta-analysis. Additionally, all the included researches were retrospectively investigated and may risk intrinsic structural biases. Moreover, findings concerning the biological underpinnings of TLS were still at the speculative and analytic stage based on gene signature, without in vivo and in vitro functional validation.

Future studies should focus on establishing a common standard for identifying and quantifying TLS and future prospectively validating it in randomized trials for better clinical applications. Second, pinpointing dynamic changes of cellular components and location within TLS during immunotherapy leads to better comprehending its biological implications. Moreover, inducing the formation of TLS, like by intratumoral injection of vital cytokines such as CXCL13 (Delvecchio et al. 2021), administration of engineered cells (GeurtsvanKessel et al. 2009) and tumor vaccines (Zhang et al. 2021), may provide a neoteric perspective for synergistic immunotherapeutic method.

## Conclusions

In brief, higher TLS levels positively correlate with higher immunogenicity and immunoactivity in TME, demonstrating potential in predicting postoperative prognosis and immunotherapy response of GC. Future studies with prospective design are needed to validate the clinical significance of TLS, individually or jointly with other markers, across different cancer types.

### Supplementary Information

Below is the link to the electronic supplementary material.

Supplementary Supplementary table 1. Searching strategy in public databases of meta-analysis. file1 (DOCX 16 KB)

Supplementary Supplementary table 2. Newcastle-Ottawa Quality Assessment Scale evaluating the quality of the included studies. file2 (DOCX 19 KB)

Supplementary Supplementary table 3. Details of covariates used for multivariate analysis of the included studies file3 (DOCX 17 KB)

Supplementary Supplementary figure 1. Meta-analyses investigating the associations between tertiary lymphoid structures (TLS) levels and overall survival of gastric cancer after radical resection. Forest plots demonstrating the results in the univariate analysis (A-C), multivariate analysis (B-D), hematoxylin-eosin (H&E) staining detection (E), H&E and immunohistochemistry staining detection (F), sample size ≥ 200 (G), sample size < 200 (H), median age ≥ 60 (I), and median age < 60 (J) subgroups. Each result was displayed by the hazard ratio with corresponding 95% confidence interval. file4 (PDF 266 KB)

Supplementary Supplementary figure 2. Publication bias and sensitivity tests of meta-analyses. The funnel plot examined the publication bias (A). Trim-and-fill method tested and adjusted the publication bias (B-C). Sensitivity analysis as evaluated by the leave-one out-test (D) file5 (PDF 152 KB)

Supplementary Supplementary figure 3. The tertiary lymphoid structures (TLS)-level profiles of different molecular subtypes of gastric cancer (GC) and their prognostic effects. TLS levels varied significantly among different molecular subtypes of GC (A). Overall survival differences between high/low TLS-level groups of EBV-infected (B), genomically stable (C), microsatellite instability (MSI)-H (D), and chromosomally unstable (E) of GC file6 (PDF 225 KB)

## Data Availability

Data used to support the findings of this study are available from the corresponding author upon reasonable request.

## Abbreviations

BCR: B-cell receptor
CI: Confidence interval
DGC: Diffuse gastric cancer
EBV: Epstein-Barr virus
GC: Gastric cancer
H&E: Hematoxylin–eosin
HR: Hazard ratio
ICI: Immune checkpoint inhibitor
IHC: Immunohistochemistry
MSI: Microsatellite instability
MC: Mast cell
NOS: Newcastle–Ottawa Scale
OR: Odds ratio
OS: Overall survival
PD: Progressive disease
PR: Partial response
ssGSEA: Single-sample gene set enrichment analysis
SD: Stable disease
TCR: T-cell receptor
TLS: Tertiary lymphoid structure
TMB: Tumor mutation burden
TME: Tumor microenvironment
