# Supplementary material for: Analyzing the associations between tertiary lymphoid structures and postoperative prognosis, along with immunotherapy response in gastric cancer: findings from pooled cohort studies
Source: J Cancer Res Clin Oncol. 2024 Mar 22;150(3):153. doi: 10.1007/s00432-024-05672-y (PMC10959798; doi:10.1007/s00432-024-05672-y)
Supplement: Supplementary file 1 — Supplementary Supplementary table 1. Searching strategy in public databases of meta-analysis. file1 (DOCX 16 KB) [file 432_2024_5672_MOESM1_ESM.docx]

**Supplementary table 1. Searching strategy in public databases.**

| **Databases** | **Searching string** | **Number of retrieved articles** |
| --- | --- | --- |
| PubMed | (((Stomach Neoplasms[MeSH Terms]) OR (Gastric Neoplasms[MeSH Terms]) OR (Gastric Cancers[MeSH Terms]) OR (stomach cancers[MeSH Terms]) OR (Cancers, Stomach [MeSH Terms]) OR (Neoplasms, Gastric [MeSH Terms])) AND ((Tertiary Lymphoid Structure[MeSH Terms]) OR (Tertiary Lymphoid Organ[MeSH Terms]) OR (Ectopic Lymphoid Organ[MeSH Terms]) OR (Tertiary Lymphoid Tissue[MeSH Terms]) OR (Ectopic Lymphoid Follicle[MeSH Terms] OR (Ectopic Lymphoid Like Structure[MeSH Terms]) OR (Lymphoid Structures, Tertiary[MeSH Terms]))) | 35 |
| Cochrane Library | ((Neoplasm):ti,ab,kw OR (Cancer):ti,ab,kw)) AND ((Gastric):ti,ab,kw OR (Stomach):ti,ab,kw)) AND ((Tertiary Lymphoid Structure):ti,ab,kw OR (Lymphoid Organ):ti,ab,kw) | 7 |
| Embase | 'stomach cancer'/exp AND 'tertiary lymphoid structure'/exp AND [english]/lim | 45 |
| Web of Science | (((AB=(Gastric Cancer)) OR AB=(Stomach Cancer)) OR AB=(Stomach Neoplasm)) OR AB=(Gastric Neoplasm) AND (((((AB=(Tertiary Lymphoid Structure)) OR AB=(Ectopic Lymphoid Organ)) OR AB=(Ectopic Lymphoid Follicle)) OR AB=(Ectopic Lymphoid Like Structure)) OR AB=(Ectopic Lymphoid Follicle)) OR AB=(Ectopic Lymphoid Like Structure) | 19 |
