# Supplementary material for: Analyzing the associations between tertiary lymphoid structures and postoperative prognosis, along with immunotherapy response in gastric cancer: findings from pooled cohort studies
Source: J Cancer Res Clin Oncol. 2024 Mar 22;150(3):153. doi: 10.1007/s00432-024-05672-y (PMC10959798; doi:10.1007/s00432-024-05672-y)
Supplement: Supplementary file 2 — Supplementary Supplementary table 2. Newcastle-Ottawa Quality Assessment Scale evaluating the quality of the included studies. file2 (DOCX 19 KB) [file 432_2024_5672_MOESM2_ESM.docx]

**Supplementary table 2. Newcastle-Ottawa Quality Assessment Scale evaluating the quality of the included studies.**

| **Author** | **Representativeness of the TLS cohort** | **Selection of the non-TLS cohort** | **Ascertainment of TLS** | **Accurate statement of outcome** | **Adjustments for confounders** | **Assessment of outcome** | **Adequate time for follow-up** | **Adjustments during follow-up** | **Total score** |
| --- | --- | --- | --- | --- | --- | --- | --- | --- | --- |
| Zhe Li | 1 | 1 | 0 | 1 | 2 | 1 | 0 | 0 | 6 |
| YiXin Yin | 1 | 1 | 0 | 1 | 2 | 1 | 0 | 1 | 7 |
| Niko Kemi | 1 | 1 | 0 | 1 | 2 | 1 | 1 | 0 | 7 |
| Quan Jiang | 1 | 1 | 1 | 1 | 2 | 1 | 0 | 0 | 7 |
| Jishang Yu | 1 | 1 | 1 | 1 | 2 | 1 | 0 | 0 | 7 |
| Takuya Mori | 0 | 1 | 1 | 1 | 2 | 1 | 0 | 0 | 6 |
| Na Cheng | 1 | 1 | 1 | 1 | 2 | 1 | 1 | 0 | 8 |
| Takuya Mori | 1 | 1 | 1 | 1 | 0 | 1 | 0 | 0 | 5 |
| Yoshihito Yamakoshi | 1 | 1 | 1 | 1 | 2 | 1 | 1 | 0 | 8 |
| Qing Li | 1 | 1 | 1 | 1 | 2 | 1 | 0 | 0 | 7 |
| Wenting He | 1 | 1 | 1 | 1 | 2 | 1 | 0 | 0 | 7 |

TLS, tertiary lymphoid structure.
