# Supplementary material for: Analyzing the associations between tertiary lymphoid structures and postoperative prognosis, along with immunotherapy response in gastric cancer: findings from pooled cohort studies
Source: J Cancer Res Clin Oncol. 2024 Mar 22;150(3):153. doi: 10.1007/s00432-024-05672-y (PMC10959798; doi:10.1007/s00432-024-05672-y)
Supplement: Supplementary file 3 — Supplementary Supplementary table 3. Details of covariates used for multivariate analysis of the included studies file3 (DOCX 17 KB) [file 432_2024_5672_MOESM3_ESM.docx]

**Supplementary table 3. Details of covariates used for multivariate analysis of the included studies.**

| **First Author** | **Publication year** | **Covariates used for multivariate analysis** |
| --- | --- | --- |
| Zhe Li | 2023 | tumor grade, gender, TILs, pTNM stage |
| YiXin Yin | 2023 | PD-L1 expression, pTNM stage |
| Niko Kemi | 2023 | year of surgery, age at diagnosis, sex, perioperative chemotherapy, Lauren type, radical resection, center, pTNM stage |
| Quan Jiang | 2022 | age, gender, lymphovascular invasion, grade, Helicobacter pylori, Lauren type, location, pTNM stage |
| Jishang Yu | 2022 | CEA, CA199, chemotherapy, tumor size, pTNM stage |
| Takuya Mori | 2021 | NA |
| Na Cheng | 2021 | age, gender, location, size, lymphovascular invasion, perineural invasion, histologic differentiation, WHO classification, TILs, pTNM stage |
| Takuya Mori | 2021 | age, sex, histological type, lymphatic invasion, venous invasion, CD103+ and CD8+ TILs, pTNM stage |
| Yoshihito Yamakoshi | 2021 | age, sex, histological type, lymphatic invasion, venous invasion, neutrophil-to-lymphocyte ratio, pTNM stage |
| Qing Li | 2020 | tumor size, histological grade, tumor thrombus, lymphatic metastasis, TILs, germinal center CD8+TILs, pTNM stage |
| Wenting He | 2020 | age, tumor size, vessel invasion, histological grade, WHO classification, pTNM stage |

CA199, carbohydrate antigen 19-9; CEA, carcinoembryonic antigen TILs, tumor-infiltrating lymphocytes; NA, not available.
