# Supplementary figures and images for: Analyzing the associations between tertiary lymphoid structures and postoperative prognosis, along with immunotherapy response in gastric cancer: findings from pooled cohort studies
Source: J Cancer Res Clin Oncol. 2024 Mar 22;150(3):153. doi: 10.1007/s00432-024-05672-y (PMC10959798; doi:10.1007/s00432-024-05672-y)

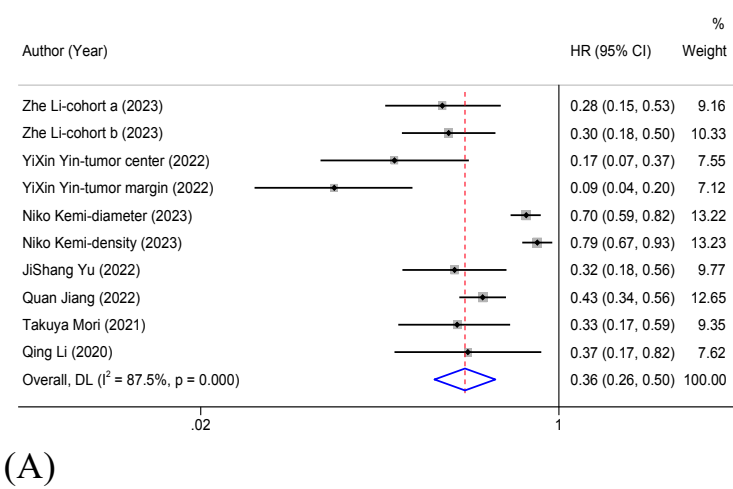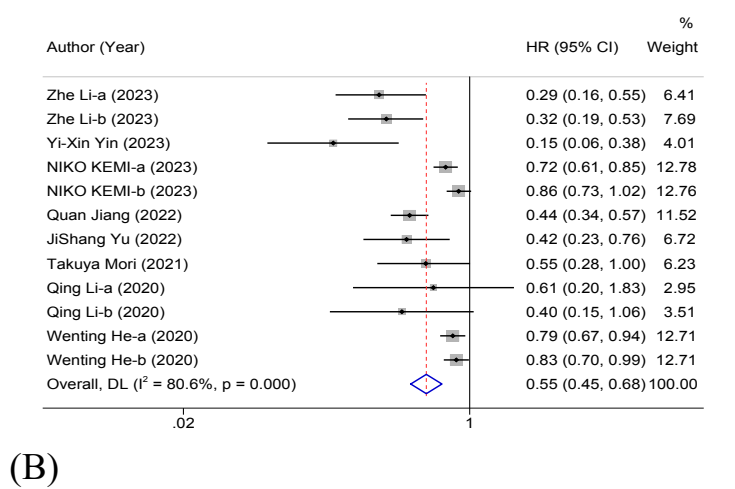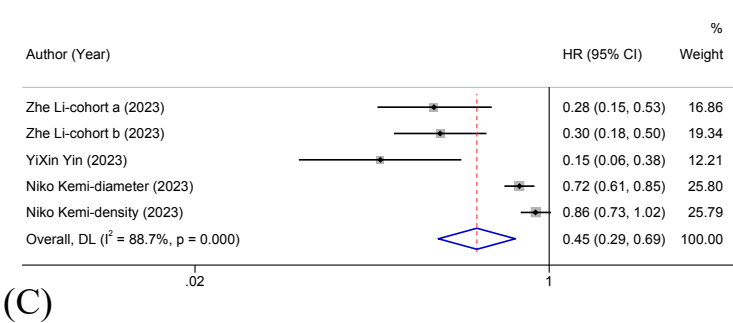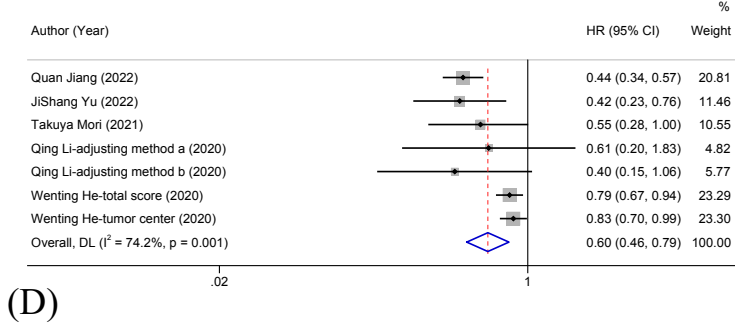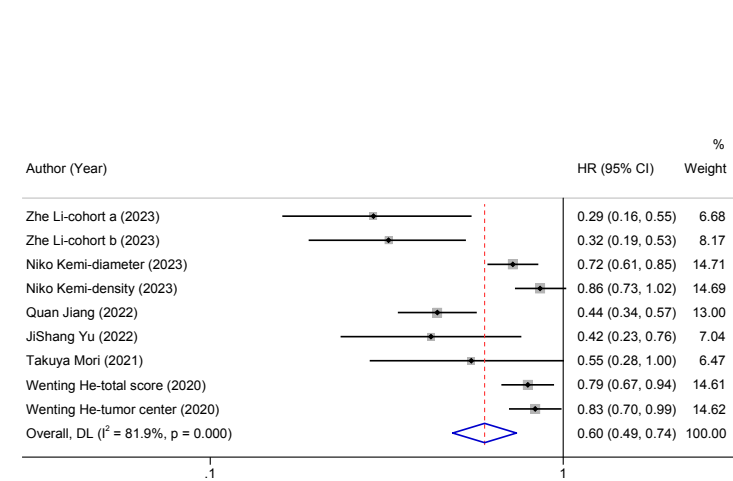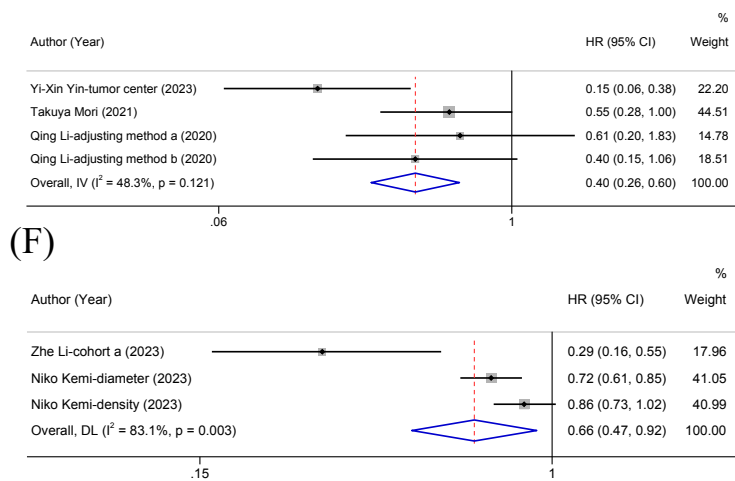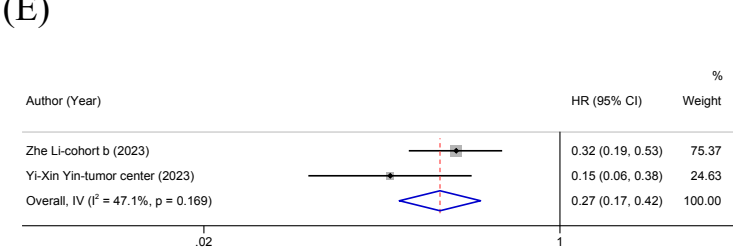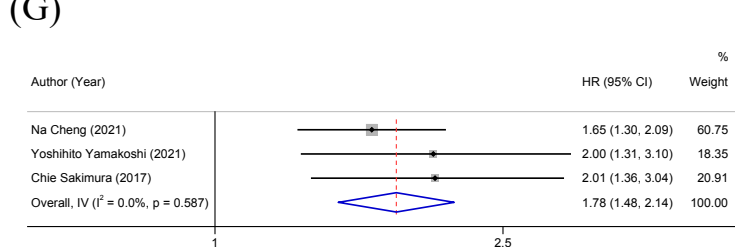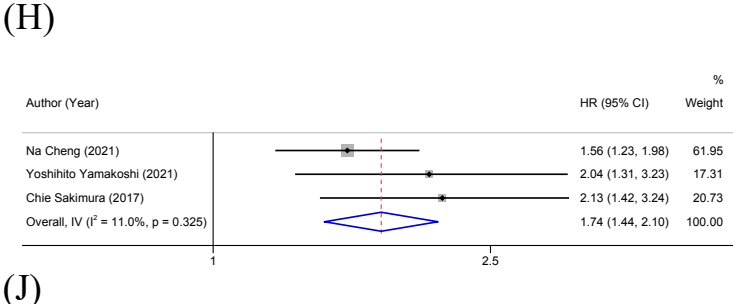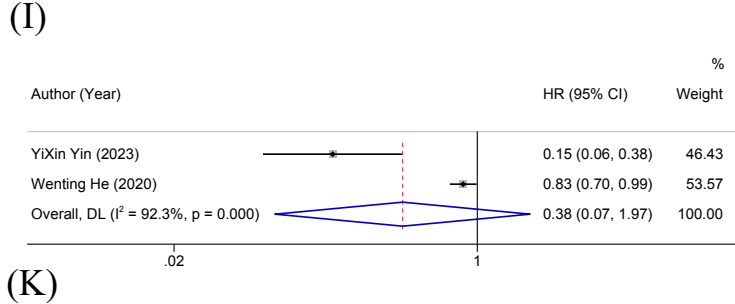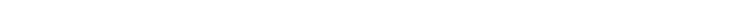

Supplement: Supplementary file 4 — Supplementary Supplementary figure 1. Meta-analyses investigating the associations between tertiary lymphoid structures (TLS) levels and overall survival of gastric cancer after radical resection. Forest plots demonstrating the results in the univariate analysis (A-C), multivariate analysis (B-D), hematoxylin-eosin (H&E) staining detection (E), H&E and immunohistochemistry staining detection (F), sample size ≥ 200 (G), sample size < 200 (H), median age ≥ 60 (I), and median age < 60 (J) subgroups. Each result was displayed by the hazard ratio with corresponding 95% confidence interval. file4 (PDF 266 KB) [file 432_2024_5672_MOESM4_ESM.pdf]

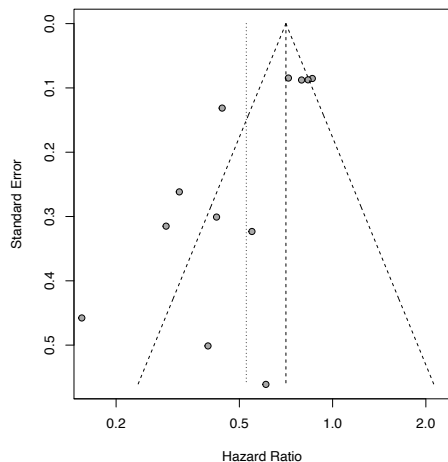

(A)

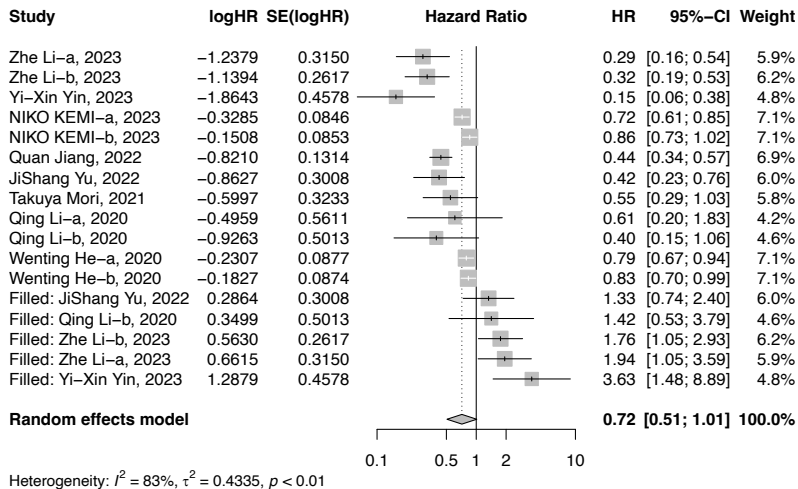

(B)

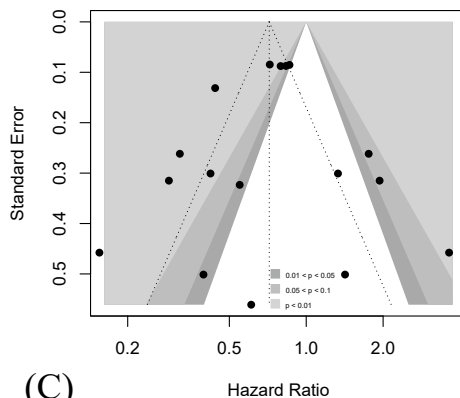

(C)

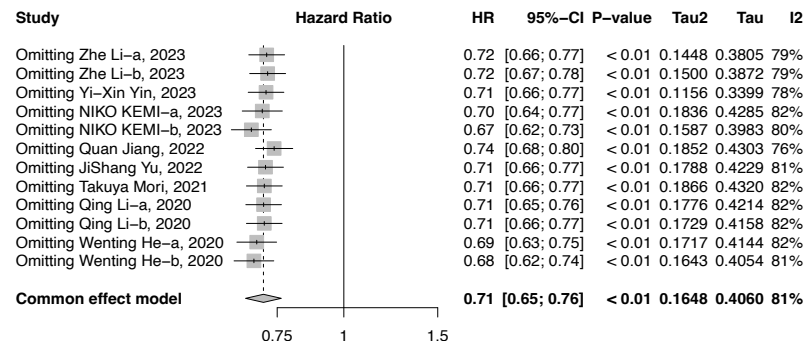

(D)

Supplement: Supplementary file 5 — Supplementary Supplementary figure 2. Publication bias and sensitivity tests of meta-analyses. The funnel plot examined the publication bias (A). Trim-and-fill method tested and adjusted the publication bias (B-C). Sensitivity analysis as evaluated by the leave-one out-test (D) file5 (PDF 152 KB) [file 432_2024_5672_MOESM5_ESM.pdf]

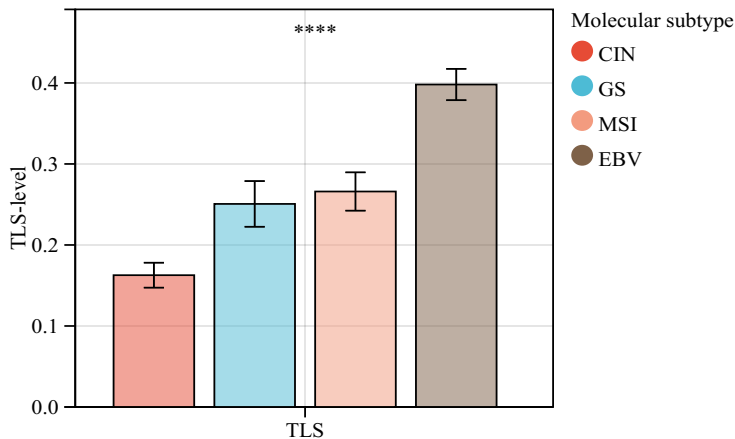

(A)

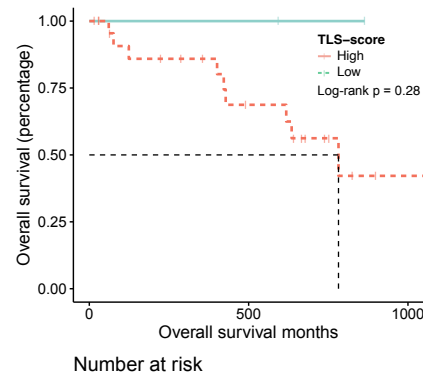

(B)

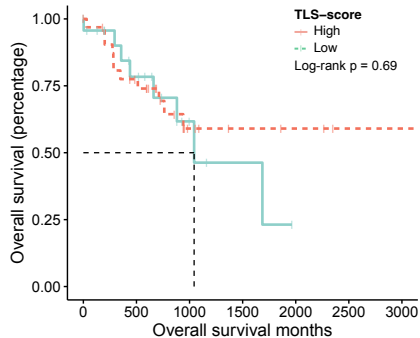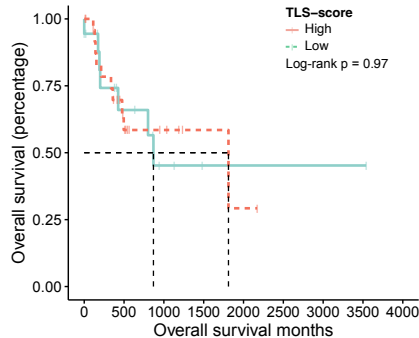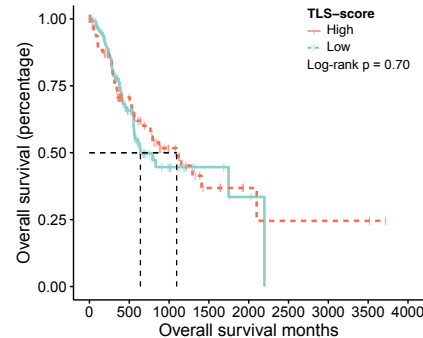

(C)

(D)

(E)

**Number at risk**

|      |    |    |   |   |   |   |   |
|------|----|----|---|---|---|---|---|
| Low  | 24 | 13 | 4 | 2 | 0 | 0 | 0 |
| High | 36 | 22 | 7 | 4 | 3 | 1 | 1 |

**Number at risk**

|      |    |    |   |   |   |   |   |   |
|------|----|----|---|---|---|---|---|---|
| Low  | 18 | 8  | 3 | 1 | 1 | 1 | 1 | 0 |
| High | 26 | 10 | 5 | 2 | 1 | 0 | 0 | 0 |

**Number at risk**

|      |     |    |    |   |   |   |   |   |   |
|------|-----|----|----|---|---|---|---|---|---|
| Low  | 123 | 47 | 15 | 5 | 2 | 0 | 0 | 0 | 0 |
| High | 82  | 42 | 19 | 7 | 3 | 2 | 2 | 2 | 0 |

Supplement: Supplementary file 6 — Supplementary Supplementary figure 3. The tertiary lymphoid structures (TLS)-level profiles of different molecular subtypes of gastric cancer (GC) and their prognostic effects. TLS levels varied significantly among different molecular subtypes of GC (A). Overall survival differences between high/low TLS-level groups of EBV-infected (B), genomically stable (C), microsatellite instability (MSI)-H (D), and chromosomally unstable (E) of GC file6 (PDF 225 KB) [file 432_2024_5672_MOESM6_ESM.pdf]
